# The Bethe-Slater curve revisited; new insights from electronic structure theory

**DOI:** 10.1038/s41598-017-04427-9

**Published:** 2017-06-22

**Authors:** R. Cardias, A. Szilva, A. Bergman, I. Di Marco, M. I. Katsnelson, A. I. Lichtenstein, L. Nordström, A. B. Klautau, O. Eriksson, Y. O. Kvashnin

**Affiliations:** 1 Faculdade de Fisica, Universidade Federal do Para, Belem, PA Brazil; 2 Department of Physics and Astronomy, Division of Materials Theory, Uppsala University, Box 516, SE-75120 Uppsala, Sweden; 3 Radboud University of Nijmegen, Institute for Molecules and Materials, Heijendaalseweg 135, 6525 AJ Nijmegen, The Netherlands; 4 Theoretical Physics and Applied Mathematics Department, Ural Federal University, Mira Str.19, 620002 Ekaterinburg, Russia; 5 Institute of Theoretical Physics, University of Hamburg, Jungiusstrasse 9, 20355 Hamburg, Germany

## Abstract

The Bethe-Slater (BS) curve describes the relation between the exchange coupling and interatomic distance. Based on a simple argument of orbital overlaps, it successfully predicts the transition from antiferromagnetism to ferromagnetism, when traversing the 3*d* series. In a previous article [Phys. Rev. Lett. 116, 217202 (2016)] we reported that the dominant nearestneighbour (NN) interaction for 3*d* metals in the bcc structure indeed follows the BS curve, but the trends through the series showed a richer underlying physics than was initially assumed. The orbital decomposition of the inter-site exchange couplings revealed that various orbitals contribute to the exchange interactions in a highly non-trivial and sometimes competitive way. In this communication we perform a deeper analysis by comparing 3*d* metals in the bcc and fcc structures. We find that there is no coupling between the *E*_*g*_ orbitals of one atom and *T*_2*g*_ orbitals of its NNs, for both cubic phases. We demonstrate that these couplings are forbidden by symmetry and formulate a general rule allowing to predict when a similar situation is going to happen. In *γ*-Fe, as in *α*-Fe, we find a strong competition in the symmetry-resolved orbital contributions and analyse the differences between the high-spin and low-spin solutions.

## Introduction

The Bethe-Slater (BS) curve^1–3^ formed an early fundament for the understanding of magnetism and magnetic ordering of the 3*d* transition metal elements. It successfully explains the antiferromagnetic (AFM) order of bcc Cr, as well as the ferromagnetic (FM) ground state of bcc Fe, hcp Co and fcc Ni^4,5^, as well as metastable polymorphs of these elements, such as bcc Co^6^ and bcc Ni (see e.g. ref.^7^ and references therein). The microscopic mechanism behind this curve is a common textbook example of direct exchange, in which a relationship between magnetic ordering and the nearest-neighbour (NN) distance between atoms (relative to the radial extent of the 3*d* wavefunction) can be derived^4,5^. The BS curve furthermore provides a practical tool to analyze the complex magnetism of elemental Mn^4,5^ and many Mn compounds, since this element is situated very close to a point of the BS curve where the AFM and FM orders are extremely close in energy. In fact, it is empirically recognized that for many Mn compounds, a critical parameter controlling the type of magnetic order is provided by the NN distance between Mn atoms^8^, where larger separations result in ferromagnetism and smaller NN distances often are connected to AFM order. In some cases the BS curve can be used to explain the temperature dependence of the magnetisation of more complex systems, such as amorphous magnets^9^. On the other hand, the existing microscopic theory behind the BS curve is too simple, and it can not, for example, explain the pressure dependence of the Curie temperature of bcc Fe^10^ or the AFM order of Fe spins on W(001) surface^11^.

With the rapid development in modern theories of electronic structure, one might ask if new information about BS curve may be obtained, as well as the origin of magnetic ordering in 3*d* elements. Nowadays one can evaluate the exchange interaction *J*_*ij*_ between atoms centred at site *i* and *j* with a good accuracy and directly from electronic structure theories using several methods^12–15^. This development, together with the accuracy in which modern electronic structure theory reproduces atomic magnetic moments (*M*_*s*_’s), allows for a parameter-free evaluation of the most important parameters of effective spin-Hamiltonians, where the Heisenberg Hamiltonian (HH) is most commonly discussed. Paired with modern theories for atomistic spin dynamics^16–18^ most of the relevant excited state properties of magnets can then be evaluated, without having to rely on experimental input.

Previously^19^, we have investigated the series of 3*d* metals in their bcc phases by performing a decomposition of the *J*_*ij*_’s onto *E*_*g*_- and *T*_2*g*_-derived orbital contributions. We found that even though the dominant exchange coupling across the series follows the BS curve, its microscopic structure is complicated, especially in Mn and Fe. In our previous work, the results were mostly discussed in view of *α*-Fe, where this procedure allowed us to disentangle Heisenberg and non-Heisenberg (i.e. double exchange-type) contributions to the magnetic interactions. In the present work, we discuss all 3*d* elements in greater detail and also compare bcc and fcc phases of some of them. A symmetry analysis is given, showing that the interactions between *E*_*g*_ and *T*_2*g*_ are in general important, except for the situations when they are forbidden by symmetry. We discuss the implications of our results in light of applications in ultra-fast spin-dynamics phenomena and atomistic spin-dynamics simulations in general. We also analyse the possibility to use the understanding provided here of interatomic exchange of 3*d* elements, to design new functional magnets, e.g. magneto-caloric materials.

## Results

### bcc lattice

**Figure 1:**
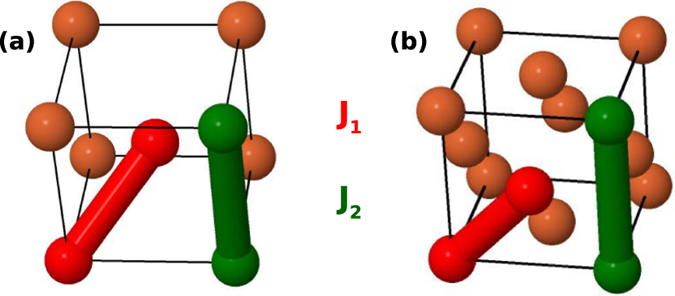
Schematic representation of the near neighbor (NN) and next-near neighbor (NNN) of a (**a**) bcc and (**b**) fcc lattice. In red the bond between NN and in green the bond between NNN.

**Figure 2:**
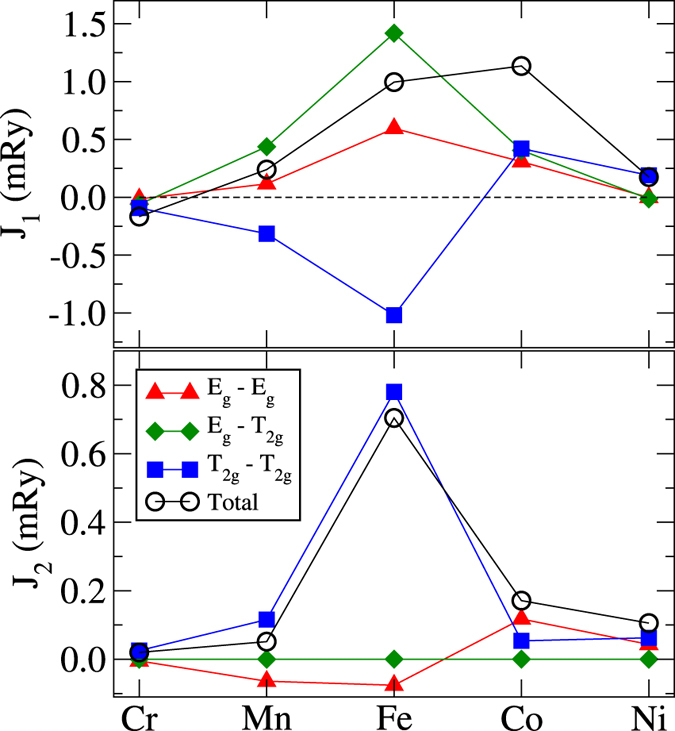
Orbitally-decomposed exchange interactions of the elemental 3*d* metals in the bcc structure. Top panel: NN coupling *J*_1_. Bottom panel: next NN coupling *J*_2_. The top panel is the same as Fig. 1 from ref.^19^, except for Cr, which was here studied for a lattice constant of 5.46 a.u., whereas *a* = 5.67 a.u. was used in ref.^19^. The results for both volumes are reported in the SM.

Interactions with the first two coordination shells play a decisive role in the formation of the Weiss field acting on *M*_*s*_. The calculated orbital-resolved NN and next NN exchange couplings (denoted as *J*_1_ and *J*_2_, respectively) in all 3*d* metals are shown schematically in Fig. 1 and results in Fig. 2. These two interactions dominate for the presently investigated systems and, as a matter of fact, most metallic magnets.

It may be seen from the figure that the total *J*_1_ coupling is larger than the total *J*_2_ one and that it follows the expected BS curve, as was already pointed out in ref.^19^. However, looking at the decomposition of each coupling into symmetry resolved contributions reveals a few interesting observations. In Cr all contributions have the same sign, corresponding to antiferromagnetism. In Mn and Fe we see a different picture: *E*_*g*_ − *E*_*g*_ and *E*_*g*_ − *T*_2*g*_ contributions are positive, while the *T*_2*g*_ one is negative, which is quite peculiar. Co and Ni atoms are usually coupled ferromagnetically, independently of their environment. From Fig. 2 (top panel) we note that for these two elements all components of the NN coupling are positive. This partially explains the robustness of their FM states with respect to e.g. the application of pressure. Recent experiments suggest that both elemental metals remain FM under extremely high compressions^20–22^.

The second NN interactions are also quite important, as shown in Fig. 2. Across the series, the largest *J*_2_ parameter is reached for Fe. An inspection of Fig. 2 (lower panel) reveals that this large *J*_2_ is dominated by the *T*_2*g*_ − *T*_2*g*_ contribution. In contrast to the NN exchange it is the *E*_*g*_ − *E*_*g*_ that is negative and the *T*_2*g*_ − *T*_2*g*_ contribution that is positive. In fact, the *T*_2*g*_ − *T*_2*g*_ contribution to the next nearest neighbour interaction is positive for all elements investigated here, whereas the *E*_*g*_ − *E*_*g*_ contribution is positive only for bcc Co and Ni. Previous work has shown in this case that an increase of the on-site correlations modifies *E*_*g*_ − *E*_*g*_ and *T*_2*g*_ − *T*_2*g*_ contributions in a different way, thus shifting the balance between the FM and AFM components^19^. The vanishing of the *E*_*g*_ − *T*_2*g*_ term is related to the fact that the corresponding six neighbours form a simple cubic structure, which possesses the full cubic symmetry. A more rigorous explanation based on the symmetry analysis will be done in Section C.

### fcc lattice

We have repeated the same set of calculations for elemental fcc metals. Since the number of neighbouring atoms is 12 in this case, while it is 8 for bcc lattice, one can expect a very different distribution of the electrons among *E*_*g*_ and *T*_2*g*_ states and, therefore, the corresponding contributions to the *J*_*ij*_’s. In contrast to the bcc phase, where all systems were calculated in their experimental volumes, the convergence of fcc metals was slightly more involved. Fe, Co and Ni were calculated in their experimental volumes. On the other hand, Mn is non-magnetic in a wide range of volumes and one has to adopt a relatively large lattice constants (*a*_*lat*_ > 7.275 a.u.) to arrive at a ferromagnetic solution^23^. In our calculation, we have adopted a lattice constant of 7.3 a.u., which is close to the minimal possible value. We proceed to the analysis of the results, bearing in mind that the results for Mn were obtained for an unphysical volume.

**Figure 3:**
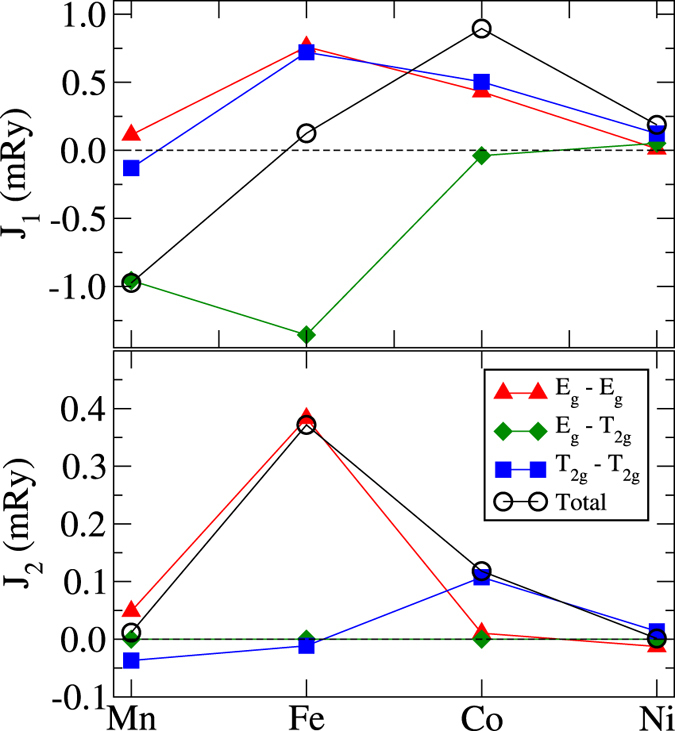
Orbitally-decomposed exchange interactions in elemental 3*d* metals in the fcc structure. The results for high-spin solution in case of Fe are shown.

Figure 3 contains the results for *J*_1_ and *J*_2_ couplings, obtained for 3*d* metals in the fcc structure. First of all, one can see that *J*_1_ again follows the BS type of curve having a maximum corresponding to Co, just as for bcc structures (Fig. 2). However, the transition from the AFM to FM coupling of the total interaction occurs later in the 3*d* series for the fcc lattice than for its bcc counterpart. In the fcc crystal structure, Fe also exhibits the most outstanding results when it comes to both *J*_1_ and *J*_2_. The *J*_1_ coupling shows the strongest competition between the ${J}_{1}^{{E}_{g}-{E}_{g}}$ and ${J}_{1}^{{T}_{2g}-{T}_{2g}}$ being FM and ${J}_{1}^{{E}_{g}-{T}_{2g}}$ which is strongly AFM and almost overweights two former contributions. Here *J*_1_ was found to be FM, but it is clear that the balance between all the contributions is very subtle. Regarding the next NN interaction, Fe is characterized by the largest *J*_2_ value as compared to other elements. This feature is found in both bcc and fcc phases. Note that the most natural phase of cobalt is hcp. Here, since the crystal symmetry (corresponding to the *D*_6*h*_ group) is lower than in the cubic lattices, the manifold of 3d orbitals is split into three irreducible representations (A_1*g*_, E_1*g*_, E_2*g*_). We analysed the orbital-resolved contributions to the *J*_1_ and *J*_2_ and found that all of them are FM. Similarly to bcc and fcc phases of Co, there is no competition between different symmetry-resolved channels.

**Figure 4:**
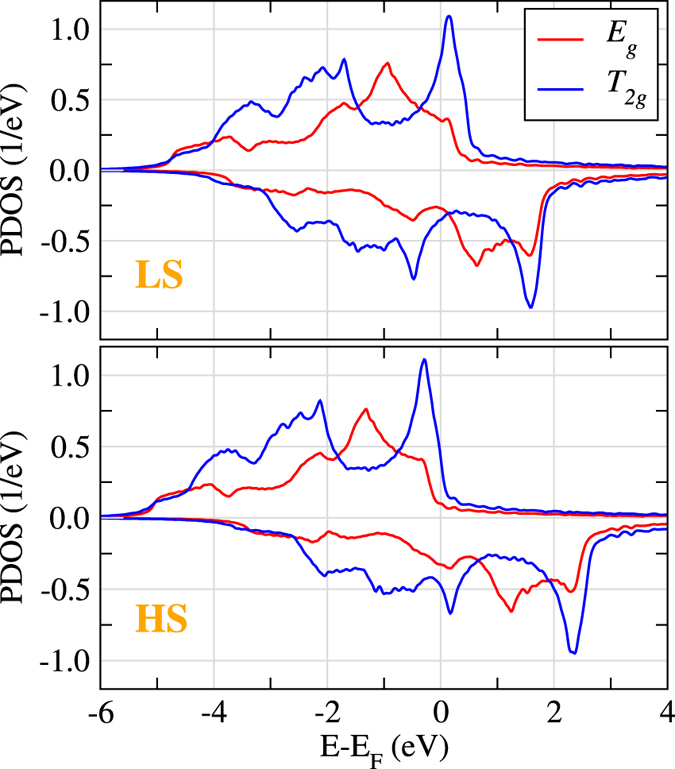
Projected density of states for the LS (top panel) and HS (bottom panel) states in fcc Fe. The results were obtained considering the experimental lattice constant of Fe in a Cu matrix (*a*_*lat*_ = 5.82 a.u.).

Figure 3 illustrates the inter-atomic exchange couplings obtained for the ground-state FM state in fcc Fe. However, *γ*-Fe can be stabilized another FM state, with a smaller moment and a higher energy. The latter is usually named low-spin (LS) solution, in contrast to the high-spin (HS) solution of the ground state. In Fe the HS and LS states are respectively characterized by a magnetic moment of 2.5 *μ*_*B*_ and 1.4 *μ*_*B*_. The density of states (DOS) of the two magnetic states is practically identical, as shown in Fig. 4. The only difference is in the exchange splitting, which is nearly two times larger in the HS configuration.

**Figure 5:**
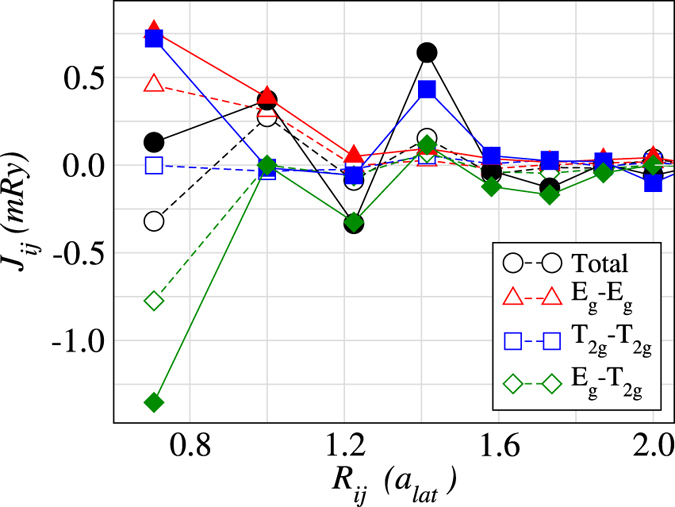
Exchange interactions in fcc Fe as a function of the distance. Dashed (solid) lines and open (filled) symbols correspond to LS (HS) state.

The *J*_*ij*_’s extracted from two magnetic states of fcc Fe are depicted in Fig. 5. The obtained magnetic interactions are long-ranged and oscillating in sign, as was already shown in refs^24^^,^^25^. The ${J}_{1}^{{E}_{g}-{T}_{2g}}$ component is AFM in both magnetic solutions and is the main source of magnetic frustration in the system. One can see that the total *J*_1_ coupling has an opposite sign in the LS state as compared with that in the HS one. The symmetry decomposition helps to understand the reasons behind this change. ${J}_{1}^{{E}_{g}-{E}_{g}}$ and ${J}_{1}^{{E}_{g}-{T}_{2g}}$ contributions get smaller, but preserve their signs, as one goes from HS to LS state. Interestingly, the ${J}_{1}^{{T}_{2g}-{T}_{2g}}$ part gets almost entirely suppressed in the LS state and it is the main driving force for the total *J*_1_ coupling to become AFM. This suppression is probably related with the fact that two pronounced peaks in the *T*_2*g*_ spin-up and spin-down DOS near *E*_*F*_ change their order in the LS state compared to the HS one (see Fig. 4), which effectively results in a decreased spin polarization.

Using the calculated *J*_*ij*_’s, we have performed atomistic spin dynamics simulations, which allowed us to predict the magnetic ground state, preferable for both magnetic solutions. For HS state we obtained a spin spiral state with ${\vec{q}}_{HS}=(0,0,1)$, which corresponds to type-I AFM state, as the lowest-energy state. The exchange integrals of the LS state suggested a non-collinear solution with ${\vec{q}}_{LS}=(0.12,0,1)$, which is incommensurate with the lattice. The latter $\vec{q}$ is very similar to the one reported experimentally for this system^26^. However, the agreement is probably fortuitous, since our calculations do not take into account the $\vec{q}$-dependence of the magnitude of the magnetic moment. The latter becomes apparent in the self-consistent spin spiral calculations, which suggest that the actual size of the moment at ${\vec{q}}_{LS(HS)}$ is somewhere in between the values we obtained for HS and LS states (see e.g. refs^27,28^). We also note that the most recent electronic structure calculations predict another configuration as the ground state at the experimental volume, i.e. a double-layer AFM state^28^. The fact that the latter is in disagreement with experiment might imply that conventional first-principles methods do not provide a proper description of the underlying electronic structure of fcc Fe and a more rigorous treatment of electron correlation effects is necessary.

### Existence of the *E*_*g*_ − *T*_2*g*_ terms

The detailed structure of the ${J}_{1}^{mm^{\prime} }$ and ${J}_{2}^{mm^{\prime} }$ matrices for selected systems is shown in the SM. As one can see in Tables S3 and S4, as well as in Fig. 2, the mixed term between two different *O*_*h*_ representations is nonzero for *J*_1_ and identically zero for *J*_2_, for all the elements. This result does not depend on the choice of the integration boundaries, i.e. the position of the Fermi level. As a matter of fact, ${J}_{2}^{{E}_{g}-{T}_{2g}}$ is exactly zero for every energy point in the integrand entering Eq. (4). Thus, these terms are likely to be forbidden by symmetry.

The property described in the previous paragraph can be explained by analysing the symmetry of the inter-site Green’s function *G*_*ij*_, entering the Eq. (4). Creating a bond between the two sites *i* and *j* in a crystal is equivalent to the symmetry-breaking of the latter and therefore the *G*_*ij*_ does not have to obey full *O*_*h*_ symmetry of the lattice. For instance, the NN bond in bcc lattice is along the (111) direction in the crystal. Such a bond remains invariant upon application of the operations belonging to the *C*_*3v*_ group. This group has less symmetry operations than the *O*_*h*_ and thus the inter-site Green’s function between NNs is expected to have less degeneracies among its elements, than any local quantity. In addition to that, the major axis of the *C*_3*v*_ group lies along (111) direction, while that of *O*_*h*_ points towards one of the Cartesian axes. Since the two axes orientations are not related by any cubic group operation, this results in the appearance of the finite elements in the *G*_*ij*_ for (111) bond, coupling *E*_*g*_ and *T*_2*g*_ orbitals. Hence, the mixed terms for the *J*_1_ coupling are allowed by symmetry in the bcc structure. Using the same reasoning, one can arrive to the same conclusions for the NN bond in the fcc lattice.

The situation is different for the next NN bonds in both the bcc and fcc structures, lying along (001), (100) and (010) directions, which obey the *C*_4*v*_ symmetry, instead. The major axis of this group coincides with that of *O*_*h*_ group and thus the same basis of cubic harmonics can be used. Here the *E*_*g*_ and *T*_2*g*_ sectors do not mix and the *J*_2_ interaction does not have the mixed contributions.

More details of the symmetry analysis and the transformation of the Green’s function can be found in the SM. It is worth mentioning that the symmetry considerations presented here are reflected in the inter-site energy integrals from the Slater-Koster tight-binding theory^29^. For an arbitrary direction of the bond in a crystal, the orbital structure of the *G*_*ij*_ is identical to that of the corresponding matrix of hoppings. Thus, Table 1 from ref.^29^ provides a direct information about which elements of *G*_*ij*_ and, therefore, *J*_*ij*_ become zero and which ones are finite.

### Long-ranged interactions

We have also investigated which orbitals participate in the long-range exchange interaction. For this purpose we have calculated the *J*_*ij*_ along a certain direction in the bcc crystal. It was shown before by Pajda *et al*.^30^ that the *J*_*ij*_’s in itinerant magnets can be very long-ranged. This is a consequence of the presence of both spin-up and spin-down electrons at the Fermi surface, which give rise to Ruderman-Kittel-Kasuya-Yosida(RKKY)-type interactions between the spins. We have verified that the bands of both spin projections cross *E*_*F*_ in all considered bcc metals, which is a result of the *sp*–*d* hybridisation.

At this point, we have to mark an important detail. In the classical RKKY mechanism, the localised magnetic moments are formed by the states, which do not implicitly interact with each other. The interaction is purely governed by the free conduction electrons. Here, in 3*d* metals, the situation is different, since it is the same 3*d* electrons which participate in the Fermi surface formation and which form the magnetic moment.

**Figure 6:**
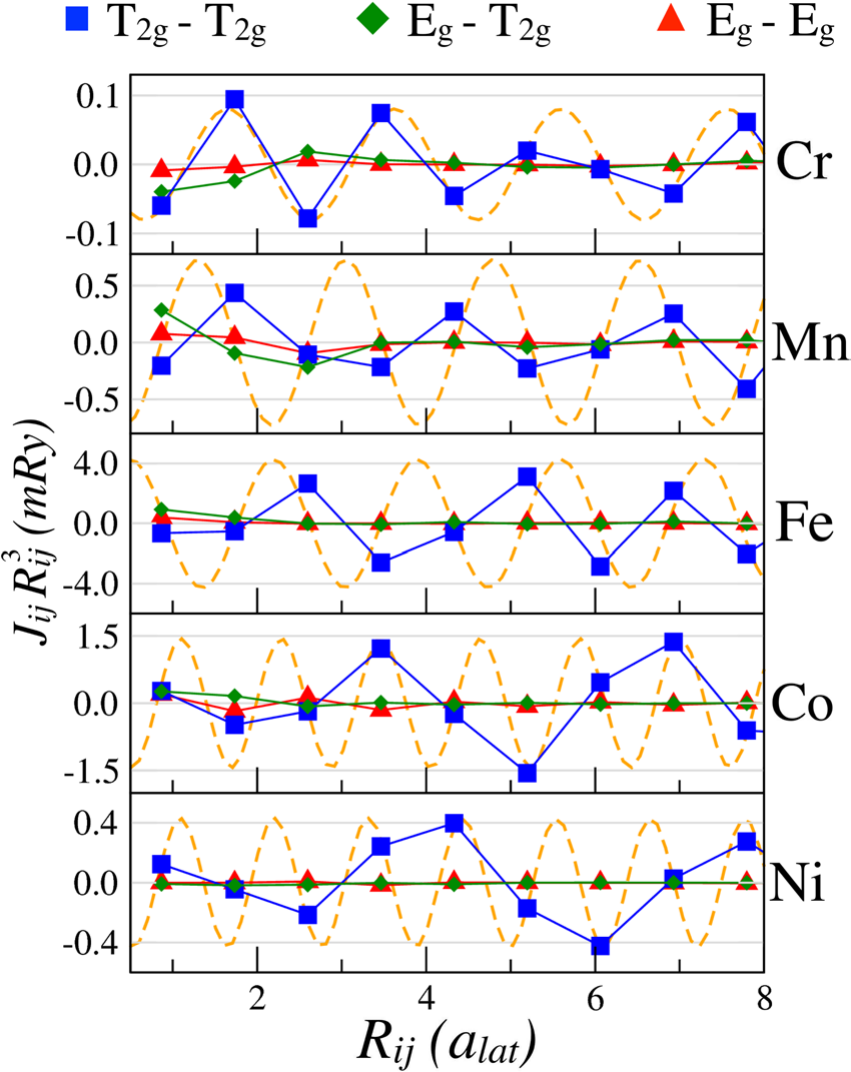
Orbitally-resolved ${J}_{ij}{R}_{ij}^{3}$ along the direction of the NN in bcc structure. Orange dashed lines show analytical functions given by Eq. (1), whose periods were obtained analysing the band structures (see text). Here, for Mn and Fe, the analytical results are scaled by a factor 1/5 on y axis for presentation purposes, such that they fit within the relevant energy window.

The calculated exchange interactions along the direction of the NN are shown in Fig. 6. First of all one can see that in all considered systems we have a well-defined long-ranged oscillatory exchange interaction. Moreover, the orbital decomposition reveals that in all systems the long-ranged behaviour is governed by the *T*_2*g*_ states. For the particular direction we have chosen, the *T*_2*g*_ states have the lobes pointing along the same axes, which might explain the predominant character of the corresponding couplings. Indeed, the situation might not hold for any general direction in the crystal. However, the considered direction is characterised by the most pronounced RKKY oscillations.

According to ref.^30^, the long-range part of the effective *J*_*ij*_’s (given by Eq. (4)) in itinerant ferromagnets has a typical RKKY-type form:

1 $${J}_{ij}{R}_{ij}^{3}={A}_{0}\,\sin \,[({k}_{F}^{\uparrow }+{k}_{F}^{\downarrow }){R}_{ij}+{\varphi }_{0}],$$

where ${k}_{F}^{\uparrow (\downarrow )}$ are the group velocities of majority (minority) spin electrons at *E*_*F*_ in the direction parallel to *R*_*ij*_, *A*_0_ and *ϕ*_0_ are some constants, defined by the electronic structure of the system. We have analysed the band structure of all considered elemental 3*d* metals. Fortunately, the topology of the Fermi surface in this BZ direction is trivial for all studied elements. Similarly to bcc Fe^19^, there is only one band per spin channel crossing *E*_*F*_. Thus, the values of ${k}_{F}^{\uparrow (\downarrow )}$ could be directly deduced from the band structure plot. The obtained values for all 3*d* metals are summarized in Table 1. Once the period of the RKKY oscillations given by Eq. (1) was identified, the predicted long-ranged dependence of the *J*_*ij*_’s was compared with the results of the electronic structure calculations. As seen from Fig. 6, the obtained sinus-functions provide an excellent qualitative explanation of the behaviour of the magnetic interactions at large distances, once again confirming the RKKY-like nature of these couplings. We note that Eq. (1) is obtained from Eq. (4) in the asymptotic limit of *R*_*ij*_ expansion. However, our results suggest that for all studied systems the RKKY-like behaviour is reached at relatively short distances. In Cr and Mn the sine-shaped fit already holds starting from *J*_1_ coupling, but in Co and Ni it matches the calculated values from two lattice constants on. The different behaviour of these metals might arise from different degree of localisation of their 3*d* orbitals.

**Table 1:** Extracted values of ${k}_{F}^{\uparrow (\downarrow )}$ (in the units of ${a}_{lat}^{-1}$) from the band structures in all considered 3*d* metals.

	Cr	Mn	Fe	Co	Ni
${k}_{F}^{\uparrow }$	1.52	2.10	2.71	3.16	3.16
${k}_{F}^{\downarrow }$	1.52	1.52	1.01	2.16	2.48

We report an interesting observation that in AFM Cr the spatial extension of the exchange interactions strongly depends on volume. For large lattice constants we obtained a relatively strong NN *J*_*ij*_’s, but farther distant couplings were found to quickly decay with increasing distance. At low volume, on the contrary, the exchange interactions show a pronounced long-range behaviour. We related these differences to the pressure-induced changes in the band structure at the *E*_*F*_ (further details can be found in the Supplementary Materials (SM)).

### Configuration dependence of the exchange parameters

At finite temperatures the *M*_*s*_’s fluctuate both in length and in direction. As a result of this, the exchange interactions in the system are expected to change with respect to their T = 0 values. In order to model the effect of transverse spin fluctuations on the *J*_*ij*_’s, we have done simulations of a large supercell where a single atomic moment was rotated with an angle *θ* with respect to the FM background. For each chosen value of *θ*, the electronic structure was calculated self-consistently and then the *J*_*ij*_-parameters were extracted, following the approach of ref.^31^.

**Figure 7:**
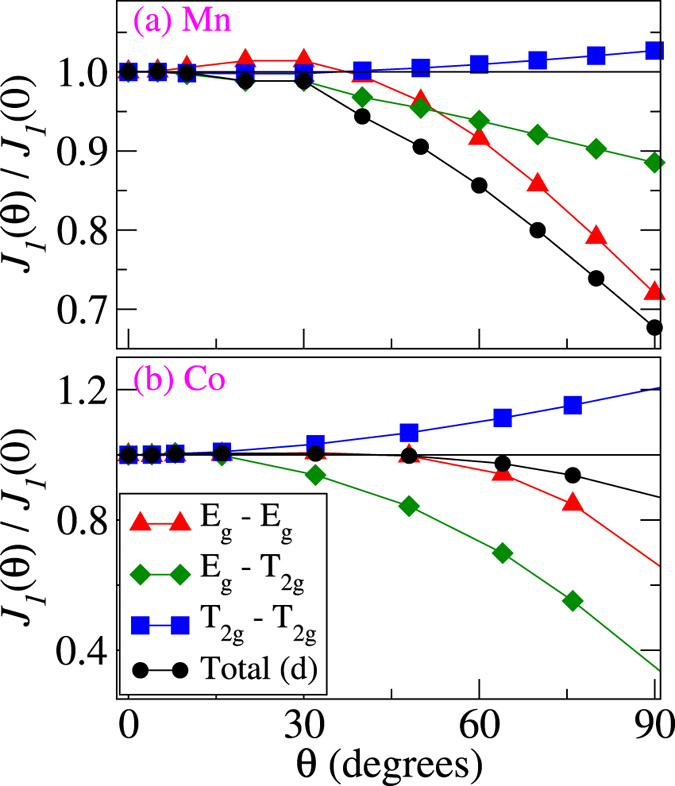
Relative change of the *J*_1_ coupling in bcc Mn (top panel) and bcc Co (bottom panel) as a function of the rotation angle *θ* of an individual magnetic moment in the FM environment. Each symmetry-resolved component is renormalised separately.

In Fig. 7 we show the calculated *θ*-dependence of the NN exchange interaction in Mn and Co. Each contribution to the *J*_1_ coupling, shown in Fig. 7, is renormalised with respect to its value at *θ* = 0. The latter corresponds to the FM state, whose data were already presented in Fig. 2. For an ideal Heisenberg magnet the coupling is supposed to be independent of the angle between the magnetic moments. The data in Fig. 7 clearly show a discrepancy from this behaviour. In both Mn and Co the total NN coupling decreases as one changes the direction of atomic spin moment at the center of the supercell. Additional information is provided by the orbital decomposition of the *J*_1_ coupling. Figure 7 clearly illustrates that the ${T}_{2g}-{T}_{2g}$ term increases in magnitude when *θ* increases, for both systems. However, in bcc Co its enhancement is almost entirely compensated by the decrease of other orbital contributions. Thus, the overall exchange coupling is found to be quite robust with respect to a variation of *θ*, as was already shown in ref.^32^. The situation is different in bcc Mn, due to the intrinsic competition between the FM and AFM ${J}_{1}^{mm^{\prime} }$ terms (see Fig. 2). Here, even though each orbital contribution shows a much weaker dependence on *θ* as compared to Co, the overall NN coupling decreases faster, because all FM contributions tend to decrease while the AFM ${T}_{2g}-{T}_{2g}$ contribution almost does not change.

A similar analysis for bcc Fe shows that the qualitative behaviour of the total *J*_1_ coupling is completely the opposite to the present case of Co: the NN coupling gets enhanced for larger *θ* values^19^. In case of Co we found that for *θ* = 90° the *J*_1_ coupling reaches only 90% of its initial value. This number is, however, closer to unity as compared to the value for bcc Fe.

**Figure 8:**
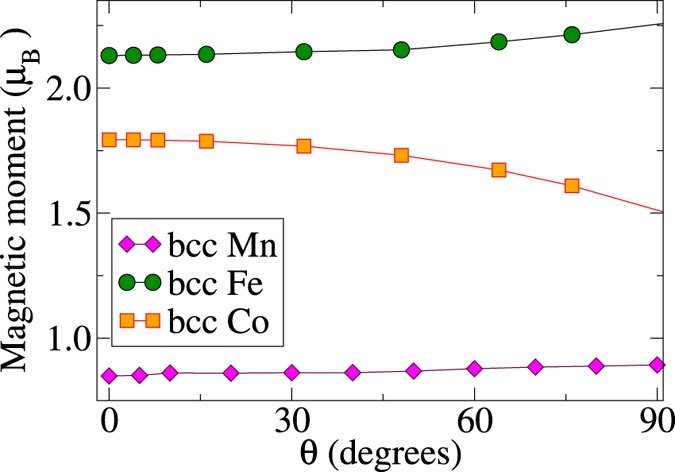
Obtained values of the rotated magnetic moment in the FM environment as a function of the rotation angle *θ* in considered bcc metals.

The latter result might be perceived as an indication that bcc Co is a more Heisenberg-like system than bcc Fe. However, such a conclusion also relies on how robust the size of the atomic magnetic moment is with respect to its rotation. In our calculations the magnitude of *M*_*s*_ for each value of *θ* was obtained self-consistently and the results are shown in Fig. 8. One can see that the rotation of a single *M*_*s*_ in Fe and Co leads to its enhancement in the former case and a decrease in the latter one. Both trends are in qualitative agreement with the results^33^ of the disordered local moment calculations, where the *average* angle between the magnetic moments was varied. Moreover, at large *θ* angles, the magnitude of *M*_*s*_ deviates stronger in Co than in Fe. Hence, at this point one can not say which of the systems is closer to the Heisenberg limit, since the modifications in *M*_*s*_ are also reflected the *J*_*ij*_-parameters.

The results for bcc Mn, shown in Fig. 8, indicate that the *M*_*s*_ value is independent on its environment. The latter usually indicates a high degree of the localisation of the magnetization density, often associated with the Heisenberg magnets. However, our results clearly show that these results are not necessarily correlated. We have shown that the *J*_1_ coupling in bcc Mn changes with *θ* quite significantly (Fig. 7(a)), so the magnetic energy of the system will deviate too, which is clearly not a Heisenberg behaviour.

## Discussion

In this article we have presented a new view on the classical BS curve, which expresses the exchange interaction as a function of the interatomic distance, considering only the change in the wavefunction overlap. By means of *ab initio* calculations, we show that the behaviour of the exchange interaction induced by a realistic electronic structure is much more complex than assumed in the BS theory. In practice each orbital at a given site is coupled to another orbital centered on a neighbouring atom and individual contributions to the exchange may have the same or opposite signs. Thus, the overall agreement between the BS curve and the total *J*_1_ coupling (Figs 2 and 3) is a result of the complicated interplay of these interactions. Our results highlight the fact that the exchange interactions in metallic systems are extremely subtle. For instance, the largest *J*_1_ value is obtained in Co, even though the ${J}_{1}^{mm^{\prime} }$ contributions are not as strong as in Fe.

As for the NN interactions in the bcc lattice, we find that the *E*_*g*_ − *E*_*g*_ and *E*_*g*_ − *T*_2*g*_ contributions are FM throughout the 3*d* series, once the *E*_*g*_ orbitals are populated. The *T*_2*g*_ orbitals can be coupled either in FM or AFM manner, since they are located at the FS and thus are sensitive to the position of the chemical potential. In particular, for Mn and Fe we find a strong competition between various ${J}_{1}^{mm^{\prime} }$ having opposite signs. On the other hand, in Cr, Co and Ni a particular sign of the magnetic interactions prevails for all orbitals, thus leaving no ambiguity for the sign of the total exchange integral. In the fcc structure, the crystal field acting on the *d* orbitals is different from the bcc lattice and hence the order of *E*_*g*_ and *T*_2*g*_ states is reverted. We find that in this environment it is the mixed ${J}_{1}^{{E}_{g}-{T}_{2g}}$ term which drastically depends on the orbital filling, whereas the other contributions to *J*_1_ show a relatively smooth dependence across the 3*d* series.

We also report a pronounced competition among the different orbital contributions to the NN interaction in fcc Fe. The bcc Fe was earlier shown to be special among other elemental metals^19^, but the magnetism of its fcc counterpart is even more complicated due to the possibility of having HS and LS solutions, which adds one more degree of freedom. Our results provide a natural explanation for the differences in the magnetic interactions observed between the two magnetic states. The *J*_*ij*_’s from LS FM state result in a non-collinear magnetic ground state, which is very similar to the one observed experimentally.

This communication also contributes to the discussion of applicability of localised models of magnetism for itinerant magnets. Usually, in order to assess the validity of the Heisenberg model for a particular system, one compares the exchange integrals extracted from different magnetic states^34,35^. Our study of configurational dependence of the *J*_*ij*_’s in bcc Co reveals an interesting observation. Even if the total exchange coupling is weakly dependent on the reference state, there are situations when this is a result of the compensation of two opposite effects: enhancement of certain orbital contributions and decrease of the others. In addition to that, the variation of the *M*_*s*_ should also be taken into account in order to complete the knowledge about the magnetic energy of the system. Thus, we conclude that in metallic systems the criterion of the applicability of the Heisenberg model can not be based only on the configurational dependence of the magnetic moments and the *J*_*ij*_’s.

Considering the atomic magnetic moment not as a single entity, but rather as a sum of contributions arising from various orbitals allows to propose new models describing magnetism of metals. In particular, disentangling ${M}_{s}^{{E}_{g}}$ and ${M}_{s}^{{T}_{2g}}$ on every atom opens a possibility to study non-coherent dynamics of these two magnetic moments. As a result of such a decoupling of symmetry-resolved contributions, one can expect the emergence of additional modes in the magnon spectra. Such phenomena might be of particular importance for the field of ultra-fast magnetisation switching^36^. Our results can be used for the extension and *ab initio* parametrisation of the atomistic spin-dynamics-based models applied to this problem^17,18,37^.

Finally, the level of modern experimental technology allows one to assemble the atomic structures in a desired way and a great effort is made in the design of new magnetic materials. The results presented in this work give new insight into the mechanisms of the exchange interactions, which opens a path for the design of magnetic materials in a more predictable way. The possibility to test the differences in symmetry-resolved exchange interactions is suggested here to be done primarily by means of optical measurements with a controlled light polarization. This will take advantage of the fact that *s* and *p*-polarized light couples differently to the orbitals of different symmetry. Thus, it should be possible to extract the information about ferromagnetic or antiferromagnetic correlations between particular orbitals from pump-probe experiments. Extracting such information is of particular importance, for instance, for Fe pnictides, which show an orbital-selective Mott transition^38^. Since spin fluctuations are suggested to be responsible for the emergence of superconductivity in these systems^39^, a detailed knowledge of the magnetic interactions between different *d* orbitals is essential.

## Methods

The electronic structure calculations were done by means of a density-functional-theory (DFT)-based computational method. All results were obtained either with a real-space linear muffin-tin orbital method within the atomic sphere approximation (RS-LMTO-ASA)^40–42^ or with a full-potential realisation of the LMTO method^43,44^. In spite of some conceptual differences in the construction of the localised basis sets, we found an excellent agreement between the results provided by both realisations of DFT. This proves that the conclusions of our orbital-resolved analysis are rather general and do not depend on the computational details. We employed standard local spin density approximation (LSDA) for the exchange-correlation energy for all the systems. The only exception was *γ*-Fe where we had to use generalized-gradient approximation (GGA)^45^, since LSDA gives too shallow total energy profile as a function of the moment^46^. Otherwise, all computational details are the same as in ref.^19^, so we redirect the reader to this work for more technical issues.

In order to extract the *J*_*ij*_-parameters, the calculations based on the magnetic force theorem^12,13^, were employed. Within this approach, the following form of the HH was parametrised:

2 $$\hat{H}=-\sum _{i\ne j}\,{J}_{ij}({\vec{e}}_{i}\cdot {\vec{e}}_{j}),$$

where ${\vec{e}}_{i}$ is a unit vector along the magnetisation at the site *i*. Within such a convention the positive sign of the *J*_*ij*_ corresponds to the FM interaction. The exchange parameter between site *i* and *j* is calculated as follows:

3 $${J}_{ij}=\frac{1}{4\pi }\Im \,{\int }_{-\infty }^{{E}_{F}}\,{{\rm{Tr}}}_{\{m\}}[{\hat{{\rm{\Delta }}}}_{i}{\hat{G}}_{ij}^{\uparrow }(\varepsilon ){\hat{{\rm{\Delta }}}}_{j}{\hat{G}}_{ji}^{\downarrow }(\varepsilon )]\,d\varepsilon ,$$

where ${\hat{{\rm{\Delta }}}}_{i}$ is the on-site exchange potential, ${\hat{G}}_{ij}^{\sigma }$ is an inter-site Green’s function (*σ* reads spin projection), *E*_*F*_ denotes the Fermi level. All terms entering this expression are matrices in orbital space. The trace is taken over the orbital indices. In most of the results shown here, only the subspace of 3*d* orbitals is taken into account for computing Eq. (4), since the contribution of *s* and *p* states does not exceed 5% of the total value of any exchange coupling.

The present article is devoted to the study of elemental transition metals and in order to study trends and changes in the electronic structure and disentangle them from the crystal structure, we investigate bcc and fcc elemental metals separately. All transition metals from Cr to Ni can be stabilised as bcc, either as the most stable allotrope or as metastable structures, grown on a suitable substrate. For the fcc lattice, we restrict ourselves to considering the same set of elements, except Cr. In these crystal structures the basis set of cubic harmonics diagonalises all local quantities, such as the site-projected Hamiltonian and, therefore, the corresponding exchange splitting (${\hat{{\rm{\Delta }}}}_{i}$). Thus, such a basis forms a natural set of physical orbitals. If the space group has a lower symmetry than cubic, it is always possible to decompose ${\hat{{\rm{\Delta }}}}_{i}$ into its irreducible representations (IR’s) and then the formalism remains the same (see e.g. ref.^47^).

Once the natural basis set is found, we can define the exchange integral between the orbital *m*_1_ on the site *i* and the orbital *m*_2_ on the site *j* as follows:

4 $${J}_{ij}^{{m}_{1}{m}_{2}}=\frac{1}{4\pi }\Im \,{\int }_{-\infty }^{{E}_{F}}\,{\hat{{\rm{\Delta }}}}_{i}^{{m}_{1}}{\hat{G}}_{ij}^{\uparrow {m}_{1}{m}_{2}}(\varepsilon ){\hat{{\rm{\Delta }}}}_{j}^{{m}_{2}}{\hat{G}}_{ji}^{\downarrow {m}_{1}{m}_{2}}(\varepsilon )d\varepsilon ,$$

The sum of all these individual orbital contributions provides the total value of the exchange integral:

5 $${J}_{ij}=\sum _{{m}_{1},{m}_{2}}\,{J}_{ij}^{{m}_{1}{m}_{2}}.$$

Using the IR’s of the *O*_*h*_ group, any exchange parameter can be written as:

6 $${J}_{ij}={J}_{ij}^{{E}_{g}-{E}_{g}}+{J}_{ij}^{{E}_{g}-{T}_{2g}}+{J}_{ij}^{{T}_{2g}-{T}_{2g}}.$$

Note that the second term in the sum contains all *mixed* (i.e. *E*_*g*_ − *T*_2*g*_ and *T*_2*g*_ − *E*_*g*_) terms between different IR’s. As we shall see, such orbital decomposition provides a lot of insightful information about the origins of the magnetic order in these systems. We have also adopted the recent generalisation of the method from refs^12,13^, allowing to deal with the non-collinear magnetic ground states^31^.

For fcc Fe, the calculated *J*_*ij*_’s were employed to parameterize the Heisenberg model, which was solved by means of atomistic spin dynamics simulations as implemented in UppASD code^48^. Exchange interactions calculated within the distance up to 3 lattice constants were used for the calculations.

## Electronic supplementary material

Supplementary Section
