# Erratum: The Bethe-Slater curve revisited; new insights from electronic structure theory

**DOI:** 10.1038/s41598-017-09611-5

**Published:** 2017-11-01

**Authors:** R. Cardias, A. Szilva, A. Bergman, I. Di Marco, M. I. Katsnelson, A. I. Lichtenstein, L. Nordström, A. B. Klautau, O. Eriksson, Y. O. Kvashnin

**Affiliations:** 1 Faculdade de Fisica, Universidade Federal do Para, Belem, PA Brazil; 2 Department of Physics and Astronomy, Division of Materials Theory, Uppsala University, Box 516, SE-75120 Uppsala, Sweden; 3 Radboud University of Nijmegen, Institute for Molecules and Materials, Heijendaalseweg 135, 6525 AJ Nijmegen, The Netherlands; 4 Theoretical Physics and Applied Mathematics Department, Ural Federal University, Mira Str.19, 620002 Ekaterinburg, Russia; 5 Institute of Theoretical Physics, University of Hamburg, Jungiusstrasse 9, 20355 Hamburg, Germany

*Scientific Reports* **7**:4058; doi:10.1038/s41598-017-04427-9; Article published online 22 June 2017

In the original version of this Article, Y. O. Kvashnin was incorrectly affiliated with ‘Faculdade de Fisica, Universidade Federal do Para, Belem, PA, Brazil’. The correct affiliation is listed below.

Department of Physics and Astronomy, Division of Materials Theory, Uppsala University, Box 516, SE-75120, Uppsala, Sweden.

This error has now been corrected in the PDF and HTML versions of the Article.

