# Supplementary material for: The Bethe-Slater curve revisited; new insights from electronic structure theory
Source: Sci Rep. 2017 Jun 22;7:4058. doi: 10.1038/s41598-017-04427-9 (PMC5481344; doi:10.1038/s41598-017-04427-9)
Supplement: Supplementary file 1 — Supplementary Section [file 41598_2017_4427_MOESM1_ESM.pdf]

The Bethe-Slater curve revisited; new insights from electronic structure theory. Supplementary materials

R. Cardias[1,2], A. Szilva[2], A. Bergman[2], I. Di Marco[2], M.I. Katsnelson[3,4], A.I. Lichtenstein[4,5], L. Nordström[2], A. B. Klautau[1], O. Eriksson[2] and Y.O. Kvashnin[2]
[1] *Faculdade de Fisica, Universidade Federal do Para, Belem, PA, Brazil*
[2] *Department of Physics and Astronomy, Division of Materials Theory, Uppsala University, Box 516, SE-75120 Uppsala, Sweden*
[3] *Radboud University of Nijmegen, Institute for Molecules and Materials, Heijendaalseweg 135, 6525 AJ Nijmegen, The Netherlands*
[4] *Theoretical Physics and Applied Mathematics Department, Ural Federal University, Mira Str.19, 620002, Ekaterinburg, Russia and*
[5] *Institute of Theoretical Physics, University of Hamburg, Jungiusstrasse 9, 20355 Hamburg, Germany*

I. VOLUME DEPENDENCE OF THE RKKY OSCILLATIONS IN AFM CR.

Here we present the results obtained for AFM Cr by varying the volume of the unit cell. We have considered two lattice parameters: 5.46 a.u. and 5.67 a.u. The former value corresponds to the experimental one and the latter was used in our previous work[1] due to convergence difficulties in RS-LMTO-ASA. Calculated long-ranged exchange interactions along the NN direction are shown in Fig. S1. As one can see, the RKKY oscillations are damped at

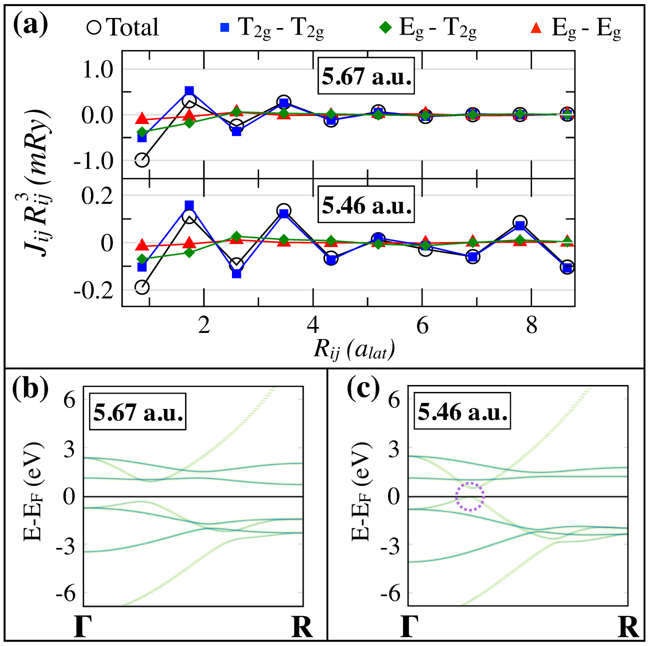

FIG. S1. Panel "a": Orbitally-resolved $J_{ij}R_{ij}^3$ along the direction of the NN in AFM Cr for two different choices of the lattice parameter. Panels "b" and "c": The corresponding band dispersions along $\Gamma - R$ direction in the BZ. Dashed circle underlines the feature which gives rise to the long-ranged magnetic couplings. Note that in this case the BZ is simple cubic, due to an AFM order.

higher volume of the unit cell, but remain well pronounced at lower ones. Quite remarkably, the period of the oscillations looks the same for both volumes. Such a different behaviour is a result of the significant changes in the band structure. It was already shown in e.g. Ref. 2 that the formation of the AFM order in Cr is accompanied by opening of the band gap. In our calculations this gap appears for both values of the lattice parameters. However, an inspection of Fig. S1(a,b) reveals that at a_{lat}=5.46 a.u. the valence band touches the E_F, which, gives rise to RKKY oscillatory exchange, according to Eq. (1) in the main text. On the other hand, at higher volumes the band dispersion is reduced and the gap completely opens. Another manifestation of this drastic change is the variation in the sublattice magnetization. For a_{lat}=5.46 a.u. the magnetic moment is about 0.65 μ_B, whereas for a_{lat}=5.67 a.u. it surges to 1.53 μ_B, which is in line with an increase of the NN J_{ij} value at larger volumes.

II. ORBITAL-RESOLVED NN EXCHANGE INTERACTIONS IN BCC TRANSITION METALS.

TABLE S1. Orbital-decomposed NN J_{ij} (in mRy) in bcc Mn, corresponding to the bond vector $\vec{R}_{ij} = (\frac{1}{2}, \frac{1}{2}, \frac{1}{2})a$.

	d_{xy}	d_{yz}	d_{xz}	$d_{x^2-y^2}$	$d_{3z^2-r^2}$
d_{xy}	-0.022	-0.032	-0.032	0.000	0.076
d_{yz}	-0.032	-0.022	-0.032	0.057	0.019
d_{xz}	-0.032	-0.032	-0.022	0.057	0.019
$d_{x^2-y^2}$	0.000	0.057	0.057	0.039	0.000
$d_{3z^2-r^2}$	0.076	0.019	0.019	0.000	0.039

TABLE S2. Orbital-decomposed NN J_{ij} (in mRy) in bcc Fe, corresponding to the bond vector $\vec{R}_{ij} = (\frac{1}{2}, \frac{1}{2}, \frac{1}{2})a$.

	d_{xy}	d_{yz}	d_{xz}	$d_{x^2-y^2}$	$d_{3z^2-r^2}$
d_{xy}	-0.098	-0.122	-0.122	0.000	0.215
d_{yz}	-0.122	-0.098	-0.122	0.161	0.054
d_{xz}	-0.122	-0.122	-0.098	0.161	0.054
$d_{x^2-y^2}$	0.000	0.161	0.161	0.282	0.000
$d_{3z^2-r^2}$	0.215	0.054	0.054	0.000	0.282

TABLE S3. Orbital-decomposed next NN J_{ij} (in mRy) in bcc Mn, corresponding to the bond vector $\vec{R}_{ij}$=(0,0,1)a.

	d_{xy}	d_{yz}	d_{xz}	$d_{x^2-y^2}$	$d_{3z^2-r^2}$
d_{xy}	0.051	0.000	0.000	0.000	0.000
d_{yz}	0.000	0.017	0.000	0.000	0.000
d_{xz}	0.000	0.000	0.017	0.000	0.000
$d_{x^2-y^2}$	0.000	0.000	0.000	0.083	0.000
$d_{3z^2-r^2}$	0.000	0.000	0.000	0.000	-0.078

TABLE S4. Orbital-decomposed next NN J_{ij} (in mRy) in bcc Fe, corresponding to the bond vector $\vec{R}_{ij}$=(0,0,1)a.

	d_{xy}	d_{yz}	d_{xz}	$d_{x^2-y^2}$	$d_{3z^2-r^2}$
d_{xy}	0.016	0.000	0.000	0.000	0.000
d_{yz}	0.000	0.332	0.000	0.000	0.000
d_{xz}	0.000	0.000	0.332	0.000	0.000
$d_{x^2-y^2}$	0.000	0.000	0.000	0.018	0.000
$d_{3z^2-r^2}$	0.000	0.000	0.000	0.000	-0.074

III. SYMMETRY ANALYSIS

Eq. (3) in the main text defines the formula used to calculate the exchange constant, J_{ij}, where one has to take the trace of the expression $\hat{\Delta}_i \hat{G}^{\uparrow}_{ij} \hat{\Delta}_j \hat{G}^{\downarrow}_{ji}$. The $\hat{\Delta}_i$ and $\hat{\Delta}_j$ matrices are diagonal matrices in orbital space. Hence, it is enough to focus on the Green's function $\hat{G}^{\sigma}_{ij}$, which is a matrix over the d-sector with orbital indices m_1 and m_2 that run over the following orbitals: d_{xy}, d_{yz}, d_{xz}, $d_{x^2-y^2}$ and $d_{3z^2-r^2}$.

We first note that the sites i and j define a "bond" in the system that can be characterized by a vector $\vec{n}_{ij}$ pointing from site i to j. Then J_{ij} has to belong to the invariant IR of the point group (PG) that consists of the subset of the crystallographic PG, in this case O_h, that preserves the bond direction $\vec{n}_{ij}$. There are four types of symmetric bond directions: 1) parallel to a cubic axis, $\vec{n}_{ij} \parallel \langle 00n \rangle$, with PG C_{4v}, 2) parallel to body diagonal, $\vec{n}_{ij} \parallel \langle nnn \rangle$, with PG C_{3v}, 3) parallel to a side diagonal, $\vec{n}_{ij} \parallel \langle nn0 \rangle$, with PG C_{2v}, and finally 4) lying in a mirror plane, $\vec{n}_{ij} \parallel \langle nnk \rangle$ or $\vec{n}_{ij} \parallel \langle nk0 \rangle$ with PG C_s (n and k are integers).

In Table S5 the IR's of $\hat{G}^{\sigma}_{ij}$ are given as obtained through subductions from the IR of O_h for the different possible PG. Since orbitals that belong to the same type of IR generally mix, we can deduce that only in the case of C_{4v}

symmetry the mixed term $J_{ij}^{E_g - T_{2g}}$ in Eq. (6) in the main text is zero. For lower symmetry directions it will be non-zero since there are contributions from the same IR for both the subduction of T_{2g} and E_g. For C_{3v} and C_{4v} our finding is consistent with the structure of Tables S1, S2, S3 and S4.

TABLE S5. Irreducible representations of the relevant subgroups of O_h obtained through subductions from O_h.

O_h	C_{4v}	C_{3v}	C_{2v}	C_s
E_g	$A_1 \oplus B_1$	E	$A_1 \oplus B_2$	$A_1 \oplus A_2$
T_{2g}	$B_2 \oplus E$	$A_1 \oplus E$	$A_1 \oplus A_2 \oplus B_1$	$2A_1 \oplus A_2$

For C_{2v}, the relevant group of e.g. the NN interactions in fcc lattice, we note that the mixing occurs only between one orbital from E_g and one from T_{2g} IR. If we specifically consider the direction [110], the two orbitals that belong to A_1 representation are $d_{3z^2-r^2}$ and d_{xy}, respectively. Then we can deduce that the corresponding Green's function matrix takes the generic form:

$$G_{ij}^{\sigma} = \begin{pmatrix} TA & 0 & 0 & 0 & MA \\ 0 & TB & TC & 0 & 0 \\ 0 & TC & TB & 0 & 0 \\ 0 & 0 & 0 & EA & 0 \\ MA & 0 & 0 & 0 & EB \end{pmatrix}, \tag{S1}$$

with the presence of MA element originating from this mixing, while the off-diagonal TC element is due to the fact that the linear combination $d_{yz} + d_{xz}$ belongs to A_2 representation and $d_{yz} - d_{xz}$ belongs to B_1. This is consistent with the actual form of the Green's function matrices obtained in the DFT calculations.

[1] Kvashnin, Y.O. *et al.* Microscopic Origin of Heisenberg and Non-Heisenberg Exchange Interactions in Ferromagnetic bcc Fe. *Phys. Rev. Lett.* **116**, 217202 (2016).
[2] Kübler, J. "Theory of itinerant electron magnetism" (OUP Oxford, 2009).
